# Supplementary material for: Assessing wildland–urban interface fire risk
Source: R Soc Open Sci. 2020 Aug 26;7(8):201183. doi: 10.1098/rsos.201183 (PMC7481699; doi:10.1098/rsos.201183)
Supplement: Supporting Information [file rsos201183supp1.pdf]

SUPPLEMENTARY INFORMATION
Communicating the Risk of Wildland Urban Interface Fires

Hussam Mahmoud and Akshat Chulahwat

July 15, 2020

1 Graph Formulation

Four layouts are tested in this study - (a) Austin (b) Jackson (c) Oakland and (d) Steamboat Springs. The details of the formulated graph for each layout are shown in Table 1. For all tests in this study, the graphs are developed using GIS data from $http://openstreetmap.org$. Each way is comprised of multiple nodes, which form the boundary of its respective way. The edge weights assigned between the nodes define the wildfire propagation probabilities from one node to another. The ways in each community are classified into different categories to recognize ignitable ways. The classification is made into categories shown in Table 2 and each of which are further classified into sub-categories to improve the accuracy of the data. Each way classification can have minor categories. For instance, type 'Building' could be further classified into - residential, religious, commercial and others. Similarly, type 'Amenity' could encompass - entertainment, financial, transportation, education and others. An important thing to note is that while all 'Building' types can be considered ignitable, not all sub-classifications of type 'Amenity' would be ignitable. A detailed list of sub-categories is listed and defined in $http://wiki.openstreetmap.org/wiki/Map\_Features$.

Table 1: Details for each community layout

Location	Total Nodes	Total Ways
Austin	11359	805
Jackson	11238	1605
Oakland	9326	845
Steamboat Springs	8068	1032

Table 2: Classification of ways based on their ignition capacity

Type	Landuse	Building	Amenity	Natural area	Leisure	Shop	Office	Route	Others
Ignition	Some	All	Some	All	All	All	All	None	None

2 Internal Propagation

To observe the effect of internal propagation on community vulnerability, a test for each selected community is conducted. In the study, all internal propagation probabilities ($P_{tr}(i,j)$) are assumed to be one, which states that if any part of a house is ignited it will lead to complete ignition. If the internal propagation probabilities $P_{tr}(i,j)$ are varied, partial ignition of houses can be modeled. A Monte Carlo simulation is conducted, where X percentage of nodes of a house are made fire resistant. The propagation probabilities from and to these particular nodes are set to be zero and for all other nodes to be one. The nodes are selected randomly at each iteration for each house and the overall community vulnerability is calculated. The results for $X = 50\%$ (i.e. half the nodes of each house are made fire resistant) for the 4 communities selected are shown below (Fig. 1). The distributions show that for the selected communities, even at $X = 50\%$, the effect of modeling partial ignition on community vulnerability is significantly low (less than 1%).

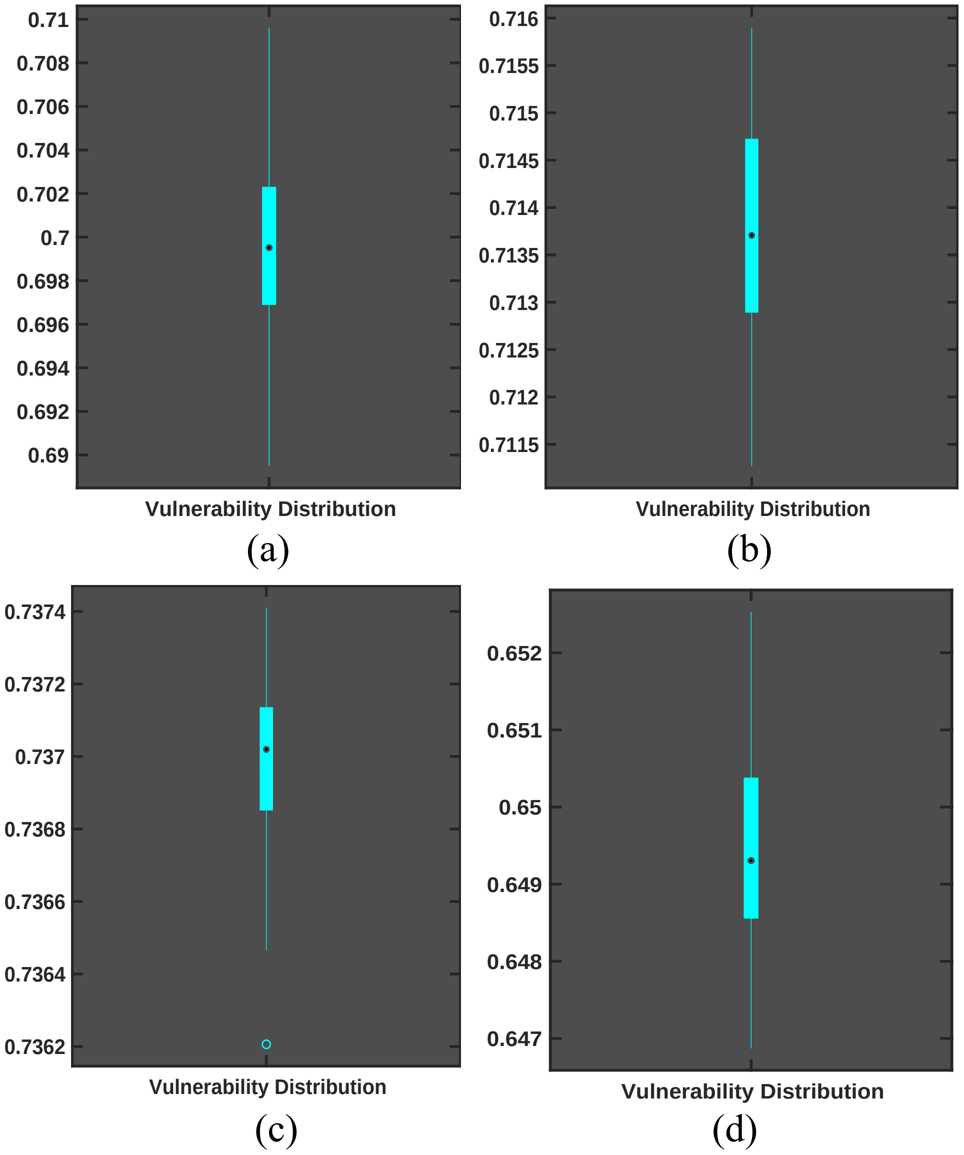

Figure 1: Box plot of vulnerability distributions for $N = 100$ iterations of (a)Austin (Texas) (b)Jackson (Wyoming) (c)Oakland (California) (d)Steamboat Springs (Colorado)

3 Vegetation Modeling

Table 3 showing distributions considered for vegetation properties. $U(a, b)$ is a uniform distribution such that a represents the minimum value and b represents the maximum value. The ignition properties for the vegetation nodes are considered to be the same as other ignitable nodes (pertaining to wooden houses), since they have similar material properties.

Table 3: Distributions for vegetation node properties

Diameter (in m)	Height (in m)
$U(2, 4)$	$U(3, 6)$

The vulnerability results for the different configurations and communities are shown in Table 4, where V_m^I is the mean vulnerability calculated when vegetation is not modeled separately and V_m^{II} is the mean vulnerability evaluated when vegetation is modeled separately for different spatial configurations of vegetation. The results for V_m^{II} show the minimum and maximum bounds obtained from 100 iterations.

Table 4: Results of Vulnerability analysis with vegetation modeling

Location	V_m^I	V_m^{II}
Austin	0.4575	(0.4432,0.4637)
Jackson	0.5225	(0.5112,0.5375)
Oakland	0.2901	(0.2821,0.3070)
Steamboat Springs	0.2017	(0.1953,0.2121)

4 Fire Intervention

In the study there are 2 types of intervention factors described – α and β (see Fig. 2). The former effects intervention strategies that occur after a house is on fire, for instance – sprinkler systems, and the latter effects the characteristics of houses that effect the probability of house becoming ignited. The intervention factor β is further formulated as shown in Eq. 1, where n are the characteristics considered of houses. Some examples of characteristics are – type of roofing material, siding material, presence/absence of decking and type, density of vegetation fuel within a certain threshold distance from house and several others. The intervention factor is described as the weighted sum of the effectiveness of each characteristic such that λ_n are the weights and p_n are the effectiveness scores associated with each characteristic. With this formulation any number of characteristics can be accommodated into the graph formulation.

$$\beta = \sum_{n=1}^{N} \lambda_n . p_n \tag{1}$$

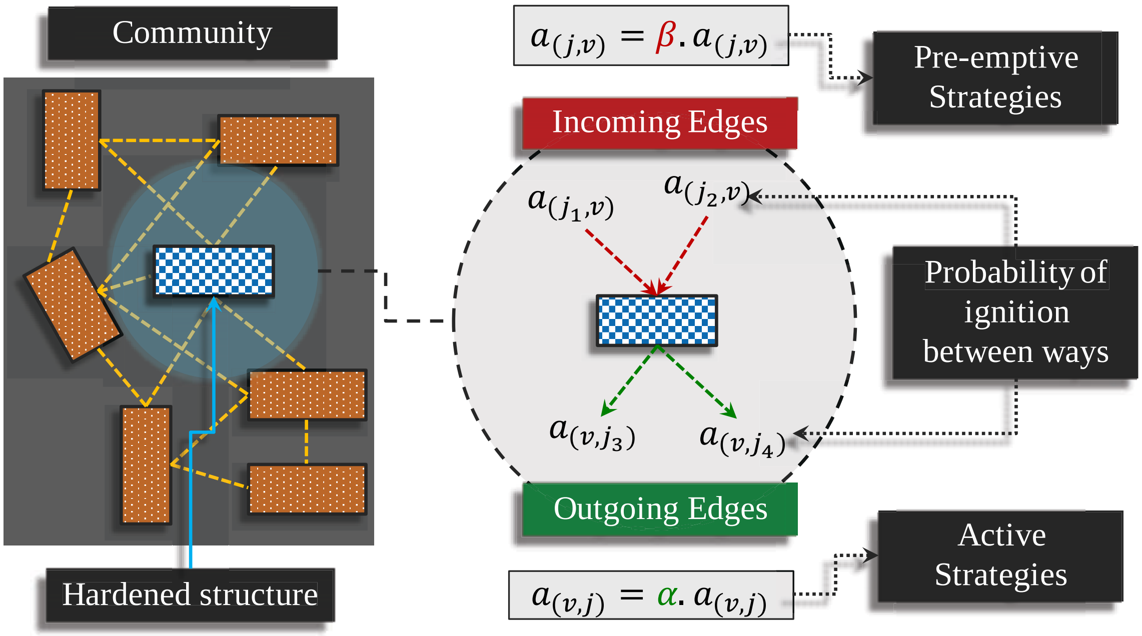

Figure 2: Fire intervention framework applied to graph model

5 Risk Framework Details

Stn ID	Stn Name	Elev	Lat	Long	Mdl	Tmp	RH	Wind	PPT	ERC	BI	SC	KBDI	HUN	THOU	TEN	STL	ADJ	IC	(Staffing Specs)
***** Alabama *****																				
10402	BROWNSBORO	529	34.7	-86.3	7G	87	39	2	.00	25	15	1	188	15	21	8	3	M	9	ERC/ 40/ 45/90/97
10702	BANKHEAD NF	989	34.3	87.3	7G	82	62	1	.00	21	16	2	158	17	20	12	2	L	4	BI / 36/ 42/90/97
10990	LIRI	1200	34.4	85.6	8E	83	53	1	.00	19	17	2	88	15	21	11	3	M	6	BI / 30/ 35/90/97
11401	ONEONTA	1104	33.9	-86.3	7G	76	79	4	.04	4	0	0	132	17	22	35	1	L	0	ERC/ 40/ 45/90/97
12201	MOUNTAIN LONGLEAF	1050	33.7	85.7	8G	71	80	6	.00	9	11	2	42	20	24	17	1	L	0	ERC/ 50/ 61/90/97
12302	TERRAPIN CREEK	805	33.8	-85.5	7G	78	62	7	.00	10	15	3	46	20	26	13	2	L	6	BI / 33/ 39/90/97
12701	TALLGA	600	33.4	86.0	7G	73	89	1	.20	1	0	0	194	19	23	35	1	L	0	BI / 33/ 39/90/97
12801	SCHOOLHOUSE	932	33.1	86.0	7G	79	71	2	.00	11	11	2	46	20	24	14	2	L	2	BI / 33/ 39/90/97
12902	SHOAL CREEK	908	33.6	85.6	7G	69	91	1	.17	7	5	1	19	19	24	21	1	L	0	BI / 33/ 39/90/97

Figure 3: Sample wildfire ignition data from WFAS Archives

The wildfire ignition probability for a particular region is calculated based on the daily data obtained from WFAS archives. The staffing specs shown in Fig. 3 is used to calibrate Eq. 8 in Materials and Methods section from the main text. The parameters - $v_u^k(d)$, $v_l^k(d)$, $p_u^k(d)$ and $p_l^k(d)$ are obtained from the data sheet such that $v_u^k(d)$ and $v_l^k(d)$ represents the upper and lower performance index and $p_u^k(d)$ and $p_l^k(d)$ are the corresponding upper and lower percentiles. A dominant performance metric each day is selected based on which the calibration parameters are opted. There are 2 key performance metrics - ERC (Energy Release Component) and BI (Burning Index). Values between stations are estimated with an inverse distance-squared technique on a 10-km grid. Once Eq. 8 is suitably calibrated, it is used to obtain the ignition probability corresponding to the observed value of the dominant performance index (I).

The second step involves calculating the vulnerability of the community in question. A graph is developed for the community layout. The wildland interface is incorporated by placing suitable source nodes in the graph. Daily Wind data is extracted from the archives of NOAA's National centers for environmental information. Based on the location, daily maximum sustained wind speed is used for evaluating vulnerability. Mean and peak wind speeds are disregarded in this study since the former would have given a conservative while the latter an overestimate. Since sustained wind speed is calculated by averaging over a 1-min period it provides a more reasonable estimate. Ideally, hourly data should be used to accurately assess changes in wind behavior for capturing wildfire behavior, however, the scope of this study is not to simulate a particular incident. Instead, the focus is to highlight the underlying patterns while evaluating wildfire risk.

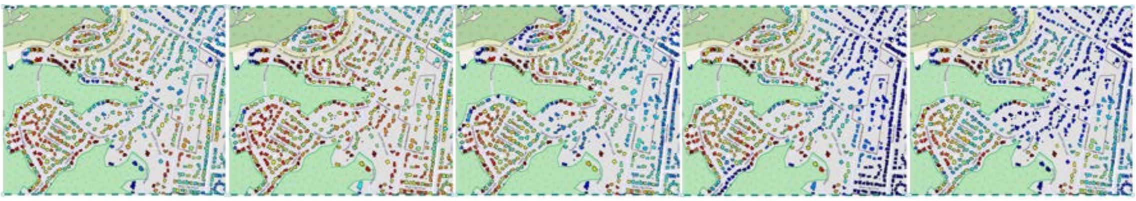

Figure 4: Vulnerability maps of 2017 August-September (left to right) for Austin (Texas)

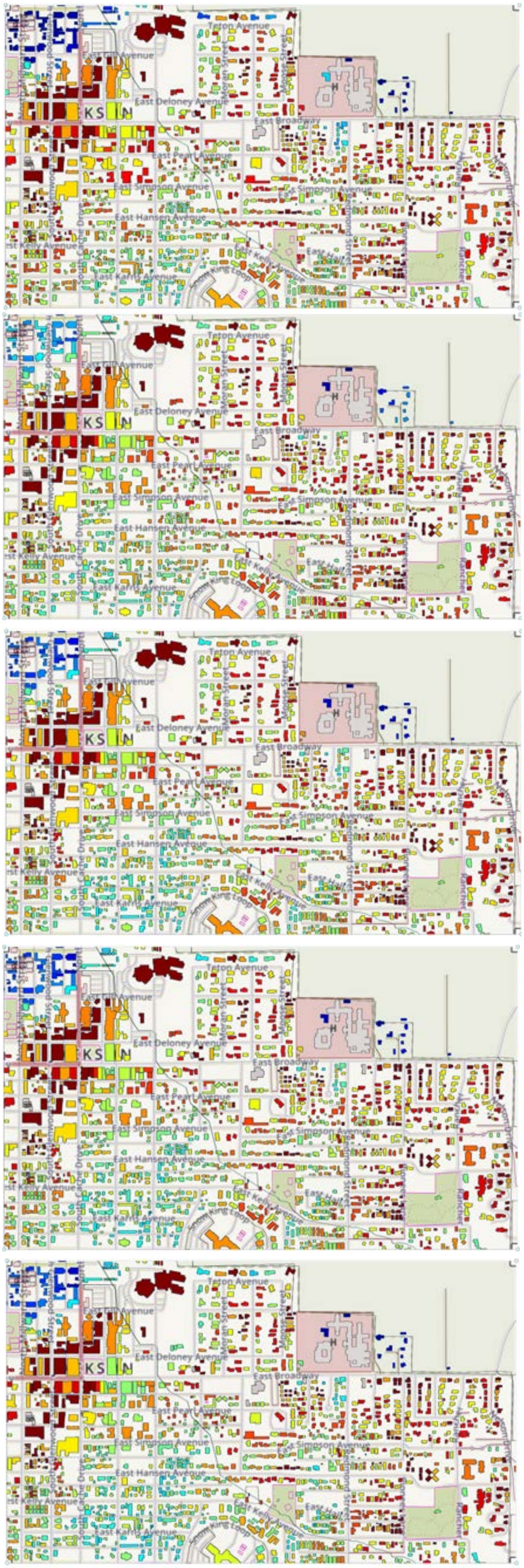

Figure 5: Vulnerability maps of 2017 August-September (left to right) for Jackson (Wyoming)

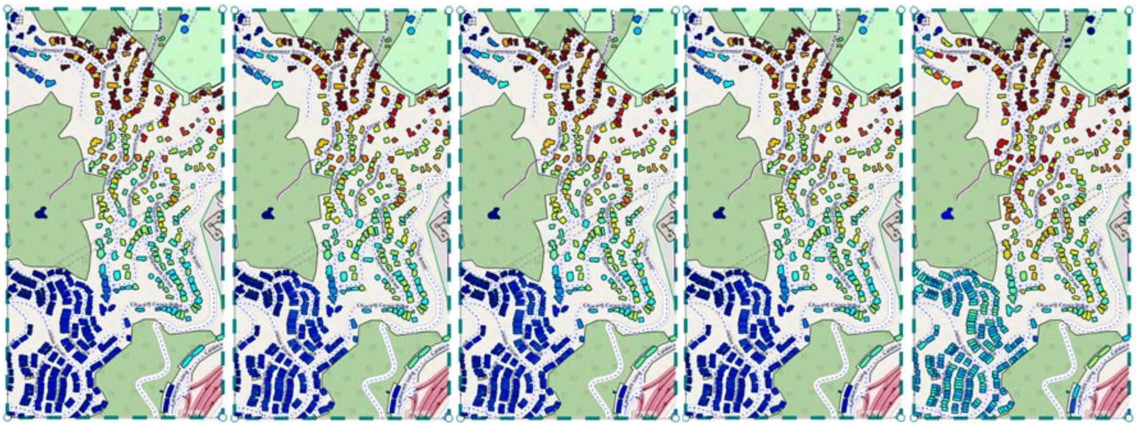

Figure 6: Vulnerability maps of 2017 August-September (left to right) for Oakland (California)

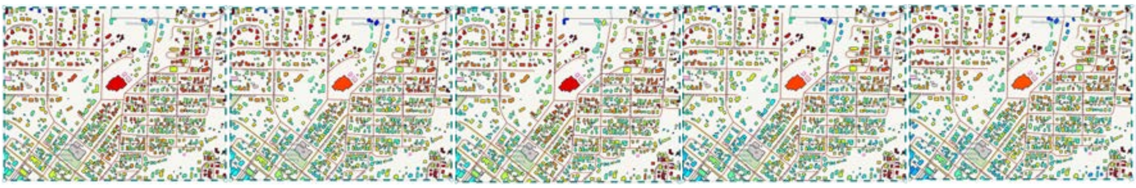

Figure 7: Vulnerability maps of 2017 August-September (left to right) for Steamboat Springs (Colorado)

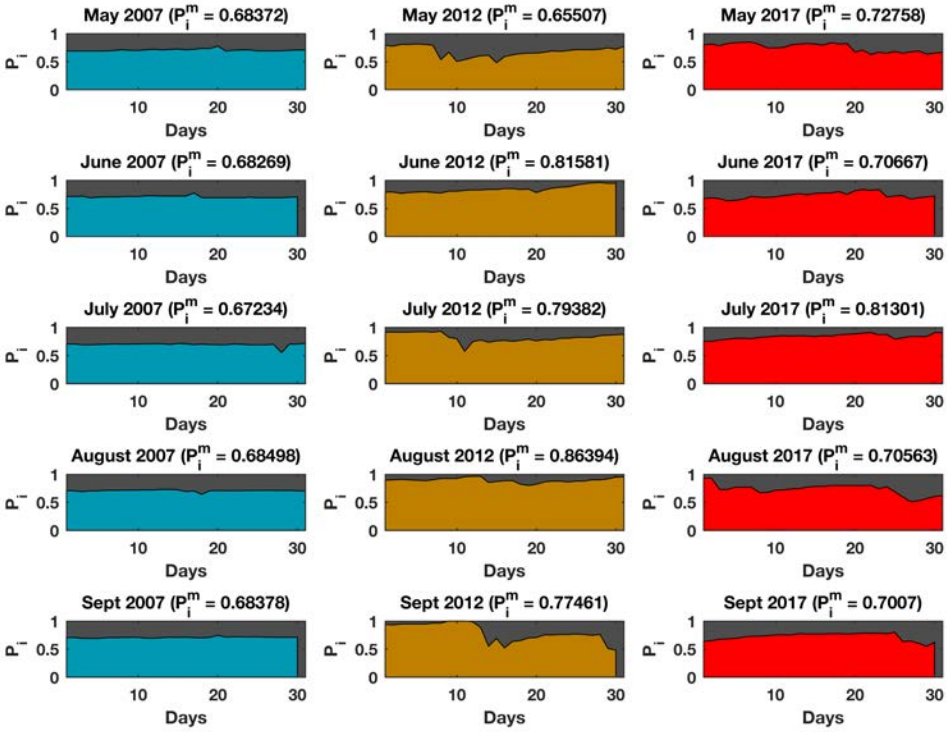

Figure 8: Probability of ignition (P_i) in months May-September for years 2007, 2012 and 2017 for Austin (Texas) (Map data ©OpenStreetMap contributors [1])

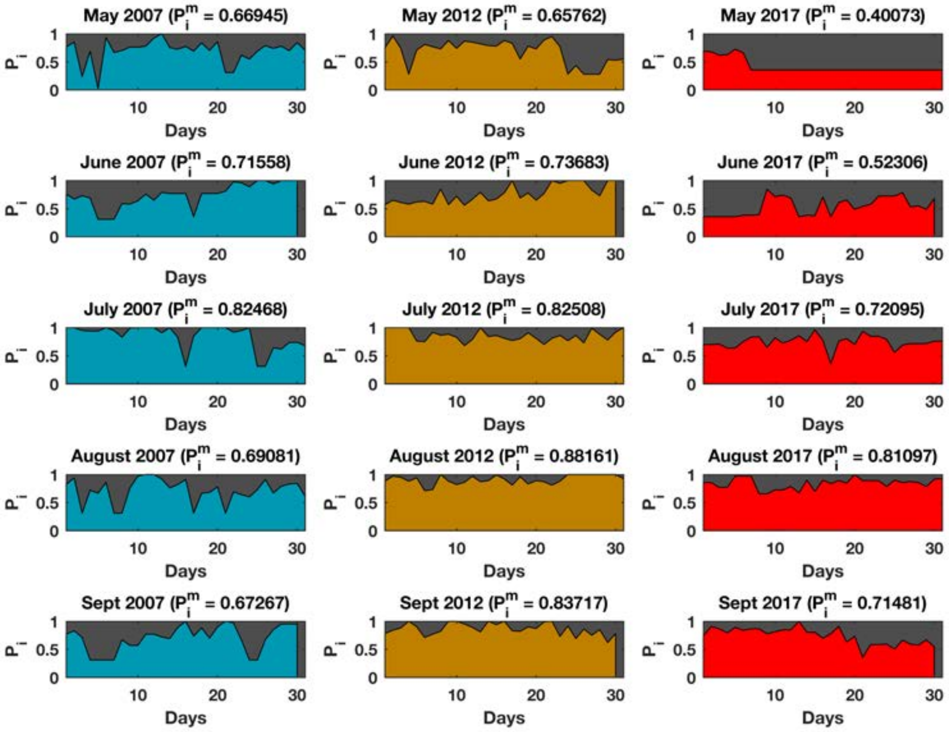

Figure 9: Probability of ignition (P_i) in months May-September for years 2007, 2012 and 2017 for Jackson (Wyoming) (Map data ©OpenStreetMap contributors [1])

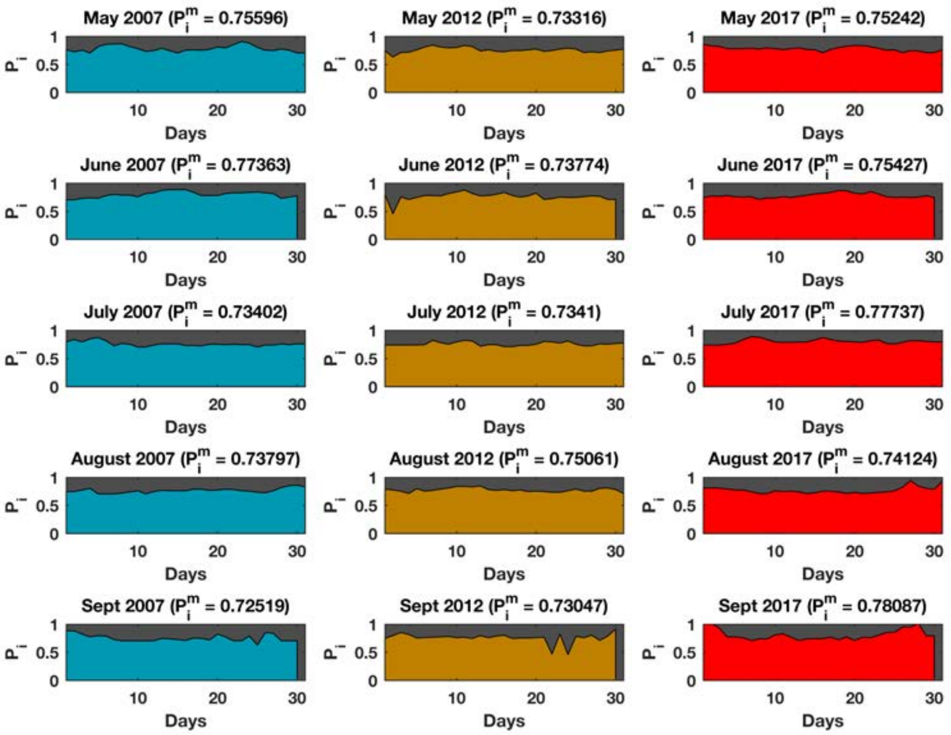

Figure 10: Probability of ignition (P_i) in months May-September for years 2007, 2012 and 2017 for Oakland (California) (Map data ©OpenStreetMap contributors [1])

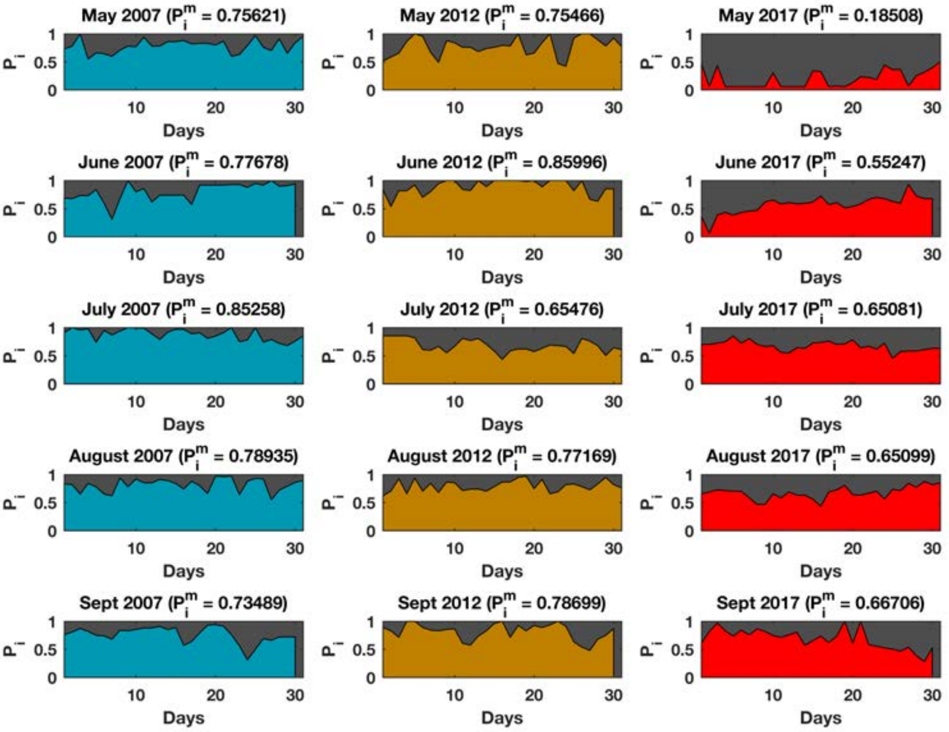

Figure 11: Probability of ignition (P_i) in months May-September for years 2007, 2012 and 2017 for Steamboat Springs (Colorado) (Map data ©OpenStreetMap contributors [1])

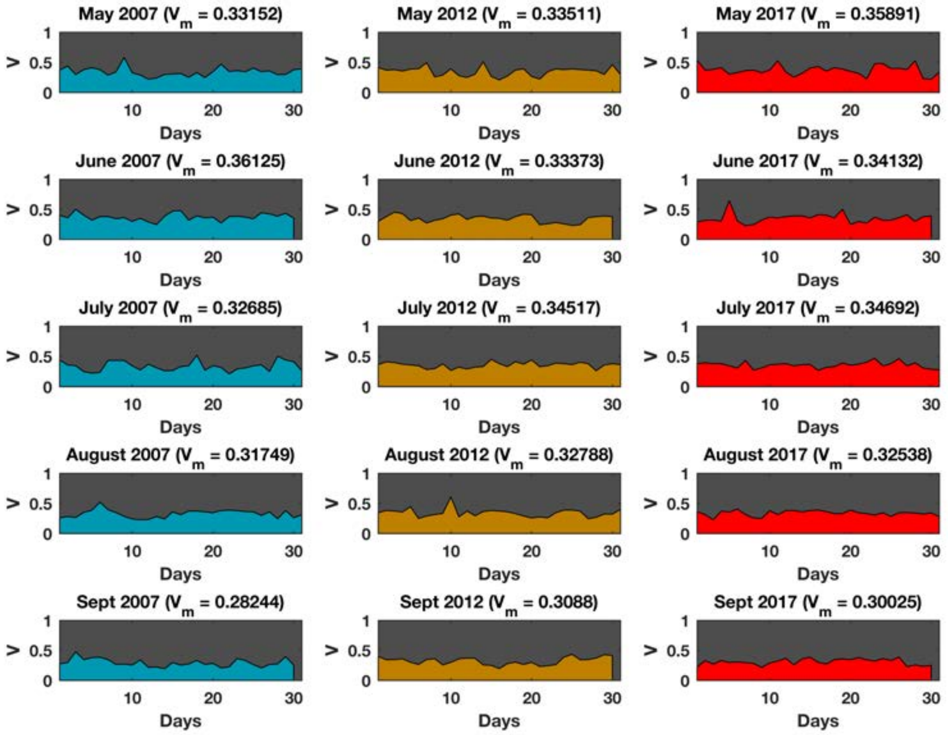

Figure 12: Community vulnerability (V) in months May-September for years 2007, 2012 and 2017 for Austin (Texas) (Map data ©OpenStreetMap contributors [1])

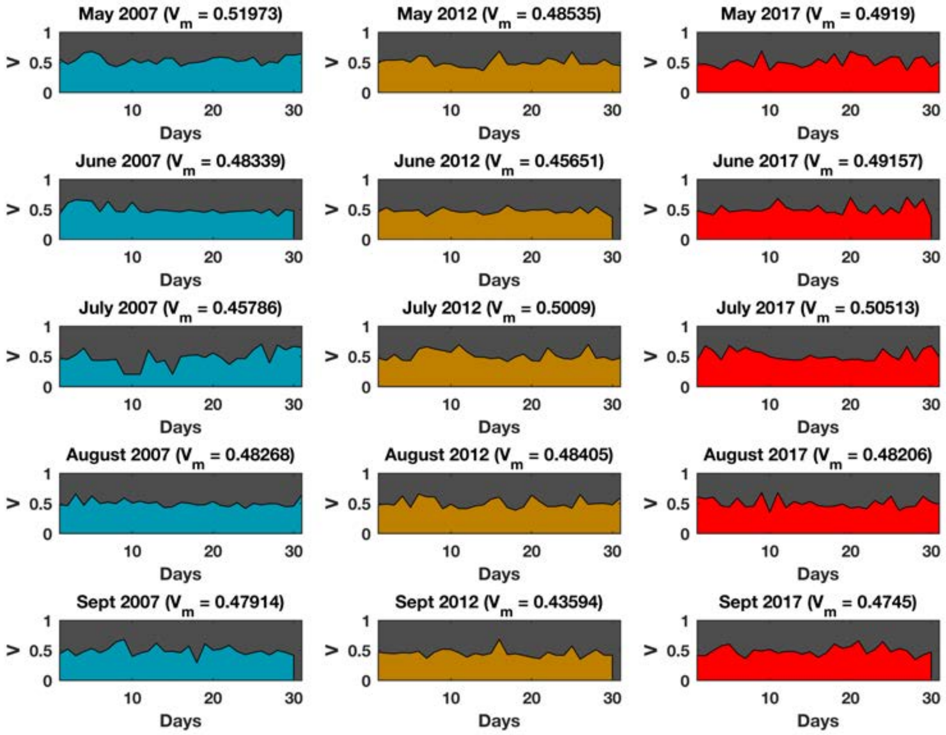

Figure 13: Community vulnerability (V) in months May-September for years 2007, 2012 and 2017 for Jackson (Wyoming) (Map data ©OpenStreetMap contributors [1])

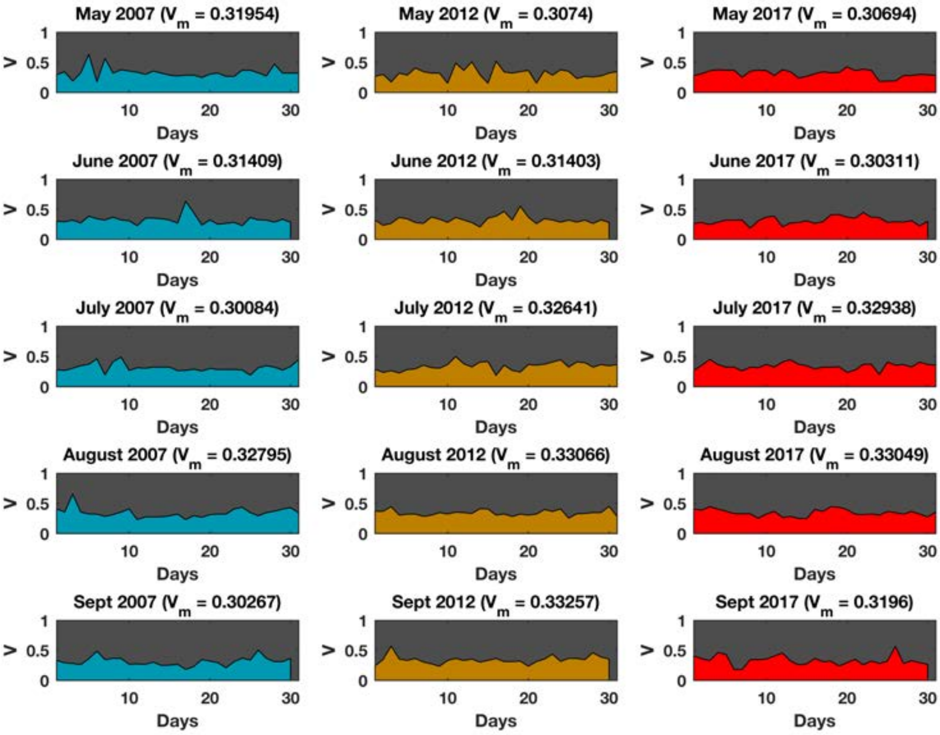

Figure 14: Community vulnerability (V) in months May-September for years 2007, 2012 and 2017 for Oakland (California) (Map data ©OpenStreetMap contributors [1])

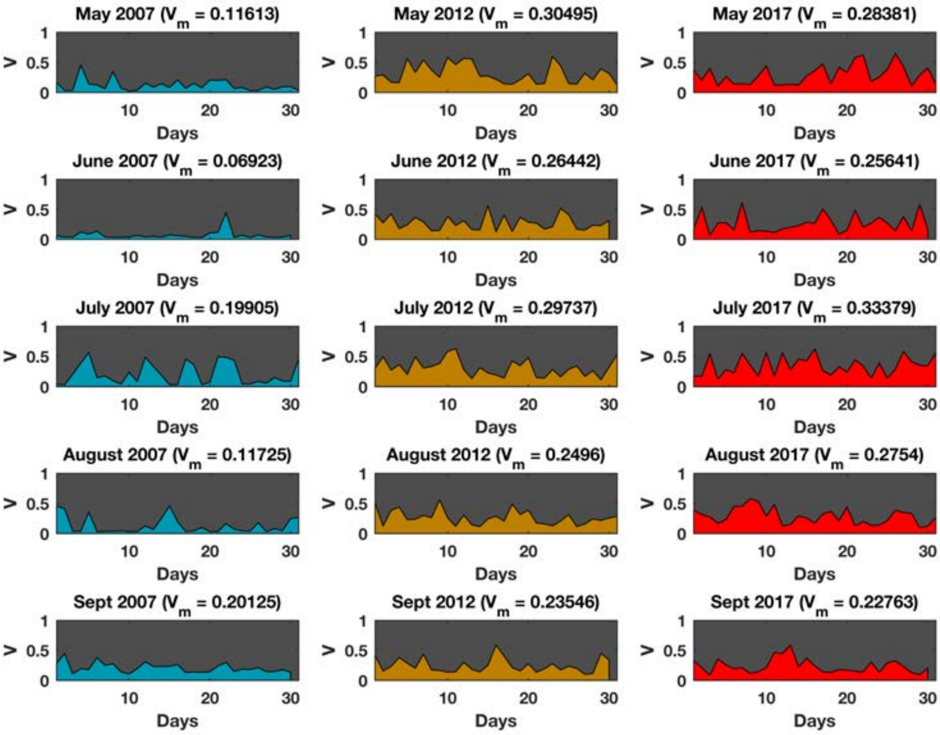

Figure 15: Community vulnerability (V) in months May-September for years 2007, 2012 and 2017 for Steamboat Springs (Colorado) (Map data ©OpenStreetMap contributors [1])

References

[1] OpenStreetMap contributors. Planet dump retrieved from https://planet.osm.org . https://www.openstreetmap.org, 2017.
